# Pathogen detection and antibiotic use in granulomatous lobular mastitis: a comparison of mNGS and culture

**DOI:** 10.3389/fcimb.2025.1570776

**Published:** 2025-06-03

**Authors:** Xu Mu, Hongmin Luo, Hanhua Li, Shenghua Chen, Yuyang Han, Lin Zhang, Wei Liu, Weilong Qiao, Shaoyi Zheng, Zhifeng Huang

**Affiliations:** ^1^ Guangdong Cardiovascular Institute, Guangdong Provincial People’s Hospital, Guangdong Academy of Medical Sciences, Guangzhou, China; ^2^ Department of Burns and Wound Repair Surgery, Guangdong Provincial People’s Hospital, Guangdong Academy of Medical Sciences, Guangzhou, China; ^3^ The First Clinical Medical School of Guangzhou Medical University, Guangzhou, China; ^4^ Department of Burns and Wound Repair Surgery, Kashi First People’s Hospital, Xinjiang, China; ^5^ Guangdong Provincial People's Hospital, Ganzhou Hospital, Ganzhou, China

**Keywords:** granulomatous lobular mastitis, pathogens, next-generation sequencing, microbial culture, sampling methods, diagnostic value

## Abstract

**Objective:**

This study aimed to evaluate the clinical microbial profile of patients with granulomatous lobular mastitis (GLM) and compare various detection methods to identify the most effective approach for pathogen detection, which could help enhance clinical diagnosis and treatment.

**Methods:**

We retrospectively analyzed data from 84 patients diagnosed with GLM, assessed the composition of pathogenic microorganisms in these patients, and compared the effectiveness of different sampling methods and detection techniques.

**Results:**

*Corynebacterium kroppenstedtii* (*C. kroppenstedtii*) was identified as the predominant microorganism among GLM patients. The positivity rate was low in skin swabs (10%) but similar in pus (40%) and tissue samples (37%). After antibiotic treatment, the pathogen detection rate of metagenomic next-generation sequencing (mNGS) (54.55%) was found to be higher than that of culture-based methods (27.27%). Among the GLM cases with pathogenic infection, although mNGS demonstrated higher sensitivity (75.0%) than culture tests (50.0%), both methods exhibited 100.0% specificity. However, the time for obtaining results with mNGS was significantly shorter (1.2 ± 0.41 days) compared to bacterial culture (5.5 ± 0.64 days) (P < 0.05).

**Conclusions:**

Our findings indicate that pus was the most suitable sample type for microbial evidence collection in patients with GLM. mNGS demonstrated superior performance compared to culture in distinguishing infectious from non-infectious cases, with reduced antibiotic interference, faster turnaround time, and higher accuracy. Based on our single-center experience, empirical cephalosporin treatment may be appropriate for these patients. Additionally, surgical intervention remains the most efficient approach for rapid and complete resolution.

## Highlights

- Pus samples should be prioritized for microbial evidence in granulomatous lobular mastitis.
- The sensitivity of mNGS technology in distinguishing infectious granulomatous mastitis is higher than that of traditional culture methods.
- mNGS has a higher pathogen detection positive rate than culture, is less affected by antibiotics, has a broader detection range, and a shorter time.
- Based on our experience, cephalosporins provide good therapeutic results, and surgery is the best option for rapid resolution.

## Introduction

Granulomatous lobular mastitis (GLM) was first described as granulomatous mastitis (GM) by Kessler and Wolloch in 1972 (Kessler and Wolloch, 1972), and as research on this condition has advanced, multiple perspectives have emerged regarding its definition. For instance, in 1987, Going et al. emphasized the histological characteristics of the disease and proposed the term GLM to highlight its unique pathological features (Going et al., 1987). Subsequently, in 1994, Donn et al. introduced the concept of idiopathic granulomatous mastitis (IGM) to emphasize the disease’s unknown etiology (Donn et al., 1994). In 2010, Boarki and Labib integrated these perspectives by defining idiopathic GLM (IGLM) (Boarki and Labib, 2010), and in 2021, the International Multidisciplinary Consensus on the Treatment of Granulomatous Lobular Mastitis recommended using a broad definition for GLM to encompass its diverse presentations (Yuan et al., 2022).

The incidence of GLM has increased significantly in recent years, accounting for 0.3%–1.9% of all breast diseases worldwide (Patel et al., 2010). This condition is particularly prevalent in Mediterranean regions and developing countries in Asia, where it represents 4%–5% of all benign breast lesions (Sheybani et al., 2015). Clinically, patients typically present with a breast lump, which may be accompanied by redness, swelling, and pain in the early stages. As the disease progresses, it can evolve into an abscess, which may rupture, forming ducts or fistulas and ultimately leading to breast deformity.

In current literature, increasing evidence suggests that the etiology of GLM is closely associated with immune dysregulation and inflammatory responses (Sheybani et al., 2016; Yaghan et al., 2019; Koksal et al., 2020). Histopathological findings support the hypothesis that antigen–antibody immune mechanisms may contribute to disease development (Kessler and Wolloch, 1972). Although various bacterial species have been detected in GLM lesions, *C. kroppenstedtii* remains consistently identified as the predominant pathogen (Yu et al., 2016; Li et al., 2022). Recent studies indicate that its pathogenic role may be associated with interactions with inflammatory mediators, potentially triggering autoimmune responses (Bercot et al., 2009; Wang et al., 2024). These findings underscore the importance of addressing bacterial infection when managing GLM.

Despite the known association with *C. kroppenstedtii*, data on the microbial composition of GLM lesions remain limited. Additionally, the diagnostic performance of current pathogen detection methods, including metagenomic next-generation sequencing (mNGS), has not been comprehensively evaluated. Therefore, to address this gap, our present study aims to investigate the pathogen spectrum in GLM patients, compare the positive detection rates of different sampling strategies (skin swabs, pus, tissues), and assess the diagnostic performance of mNGS. By developing a more accurate and efficient pathogen detection approach, this study’s findings provide clinical insights that could be used as a reference to improve the microbiological diagnosis of GLM and potentially inform individualized treatment strategies.

## Method

This retrospective study was conducted on non-lactational mastitis patients who were admitted to the Wound Repair Department of Guangdong Provincial People’s Hospital between June 2016 and June 2024. A total of 124 patients were identified, among whom 84 were diagnosed with GLM and included in the analysis.

### Inclusion and exclusion criteria

The inclusion criteria were as follows (1): patients aged 18 years or older, (2) histopathological confirmation of GLM, (3) availability of complete clinical records, and (4) microbiological confirmation of infection. For patients who underwent mNGS, samples were collected from the same anatomical site at the same time as those used for bacterial culture to ensure consistency. Cases were excluded if they had (1) superficial tissue infections resulting from surgery or trauma, (2) lactational mastitis, (3) breast malignancies, (4) incomplete clinical data, or (5) no microbiological samples collected during the treatment process.

### Metagenomic next-generation sequencing

For each patient, 3–4 mL of pus or approximately 5–10 mg of tissue was collected for mNGS. High-throughput sequencing technology was used to analyze microbial nucleic acid sequences present in the samples, which were then compared with existing microbial nucleic acid databases to identify the pathogens. The mNGS workflow consisted of two main components: experimental procedures (wet lab) and bioinformatics analysis (dry lab). The wet lab procedures included sample preprocessing, nucleic acid extraction, library construction, and sequencing. The bioinformatics analysis involved data quality control, removal of human sequences, and alignment and identification of microbial species.

### Criteria for determining positive mNGS results

The criteria for determining positive mNGS results varied based on the type of microorganism detected. For bacteria (excluding mycobacteria), fungi (excluding Cryptococcus), and parasites, a result was considered positive if one of the following conditions was met: (a) the genome coverage of unique reads mapped to the microorganism ranked within the top 10 among similar microorganisms, and the microorganism was not detected in the no-template control (NTC); or (b) the ratio of reads per million (RPM) from the sample compared to the RPM of the NTC was greater than 10, provided that the RPM of the NTC was not zero (Zhang et al., 2024).

### Criteria for distinguishing pathogenic from commensal microorganisms

To differentiate pathogenic microorganisms from commensal flora, we used a comprehensive evaluation approach based on established pathogenicity, clinical correlation, background flora assessment, and relative abundance.

Firstly, we prioritized microorganisms that had been previously reported in the literature as being associated with GLM or breast infections. Species with a known association with GLM were considered potential pathogens. For species such as *Staphylococcus epidermidis*, which are known to be conditionally pathogenic, we evaluated their relevance by considering the specific clinical context.

Secondly, we considered microorganisms to be more likely pathogenic if the patient exhibited corresponding clinical manifestations, including abscess formation, exudation, localized inflammation, or a favorable response to antibiotic therapy. This clinical correlation helped to establish the potential pathogenic role of the detected microorganisms.

Thirdly, we carefully evaluated the presence of species typically regarded as common skin commensals, as these organisms may represent contaminants introduced during sampling. We only considered such organisms clinically significant if there was a strong correlation with the patient’s symptoms or lesion characteristics, minimizing the risk of misinterpreting contaminants as pathogens.

Lastly, although we did not set strict abundance thresholds (e.g.,>20% of total reads), we gave more consideration to organisms that consistently appeared as dominant taxa in the mNGS results. Particular attention was given when these dominant microorganisms were supported by clinical or histopathological findings, as this combination reinforced their potential pathogenicity.

Overall, this systematic approach allowed for a more accurate distinction between pathogenic microorganisms and commensal flora, ensuring that the diagnostic interpretation was both clinically and microbiologically relevant.

### Diagnostic performance analysis

To evaluate the diagnostic performance of mNGS and conventional bacterial culture for pathogen detection in GLM patients, a composite reference standard was used, which incorporated both clinical diagnostic criteria and bacterial culture results to ensure accurate assessment.

First, we conducted a comprehensive review of each patient’s medical records, which was performed independently by two clinicians experienced in managing GLM and one microbiologist, considering the clinical symptoms, physical examination findings, imaging results, and responses to empirical treatment of the patients. Based on this comprehensive assessment, the cases were classified as “infectious” if there was sufficient evidence indicating an association with bacterial infection.

Pathogen identification was primarily based on bacterial culture results, as conventional culture remains the gold standard for pathogen detection in clinical practice (Esposito et al., 2016). However, we acknowledged the possibility of contamination during sample collection, handling, media preparation, or exposure to the environment, and to address this, we did not rely only on culture results when interpreting pathogen identification. In situations where there were significant discrepancies between mNGS and culture results were observed, such as when the two methods identified different species or when one method was positive and the other negative, we applied a consensus approach, whereby the clinical team and the microbiologist collectively reviewed the findings, taking into account both molecular data from mNGS and culture-based results within the clinical context for a more accurate determination of the causative organism.

Patients were classified as having non-infectious GLM if they had no signs of systemic inflammation, such as normal white blood cell counts, C-reactive protein (CRP), procalcitonin (PCT), and fungal β-glucan levels, and if both mNGS and bacterial culture results were negative.

Using this composite reference standard, we calculated the diagnostic performance of mNGS and bacterial culture by determining sensitivity, specificity, positive predictive value (PPV), and negative predictive value (NPV) to obtain a reliable comparison of the diagnostic accuracy of mNGS relative to conventional culture, particularly in distinguishing infectious from non-infectious GLM cases.

### Clinical variables

We reviewed the electronic medical records of all enrolled patients to collect data from the day of testing, which included demographic information, obstetric history, and history of autoimmune diseases, as well as any previous use of psychiatric medications and history of blunt breast trauma. Clinical manifestations related to the breast were documented, including breast lumps, redness, pain, subcutaneous fluctuation, fistula, or sinus formation. Additionally, extramammary manifestations such as arthritis, joint pain, and erythema nodosum were recorded.

We also collected data on clinical staging according to the 2021 International Multidisciplinary Consensus on the Treatment of Granulomatous Lobular Mastitis, and categorized the patients into the self-limited phase, congestive swelling phase, abscess formation phase, or complex refractory phase. Information regarding antibiotic use within two weeks before admission and during hospitalization was also documented, as well as details on bacterial culture, including sampling time, method, duration, and results. Similarly, mNGS testing details, including sampling time, method, duration, and results, were also collected.

### Statistical analysis

Statistical analysis was performed using SPSS software version 21.0 and GraphPad Prism version 10.1.0. After assessing the normality of data distribution, continuous variables were expressed as means ± standard deviations. Comparisons between two groups were performed using the independent samples t-test for normally distributed data or the Mann-Whitney U test for non-normally distributed data. For categorical data comparisons, the chi-square test was used, while the paired chi-square test or Fisher’s exact probability test was employed to compare mNGS and culture results. Clinical sensitivity and specificity were calculated using standard formulas, and statistical significance was determined with a two-tailed P < 0.05.

## Results

### Patient characteristics

A total of 84 patients were included in this study, and their age ranged from 18 to 59 years (mean ± SD: 32.87 ± 6.03). The duration of the disease varied from 7 to 730 days (95.23 ± 124.44), and the length of hospital stay ranged from 3 to 52 days (15.15 ± 7.73). According to the clinical staging criteria proposed in the 2021 International Multidisciplinary Consensus on GLM, the distribution of patients was as follows: 2 cases (2.4%) in the self-limited phase, 3 (3.6%) in the congestive swelling phase, 38 (45.2%) in the abscess formation phase, and 41 (48.8%) in the complex refractory phase (**Table 1**).

**Table 1:** Clinical presentation of the cases.

Variable	Total (n=84)	Percentage (%)
Position		
Left	46	54.8
Right	36	42.9
Bilateral	2	2.40
Skin Ulcers		
YES	32	38.10
NO	52	61.90
Sinus/Fistula		
YES	21	25
NO	63	75
Erythema Nodosum/Joint Pain		
YES	3	3.60
NO	81	96.40
Clinical Staging		
Self-limited	2	2.40
Congestive swelling stage	3	3.60
Abscess formation stage	38	45.20
Complex refractory stage	41	48.80
Recurrence		
YES	15	17.86
NO	68	82.14

### Surgical treatment

All patients with congestive swelling, abscess formation, and complex refractory phases met the surgical indications recommended by the 2021 consensus and subsequently underwent surgical treatment. Notably, the two patients classified in the self-limited phase had previously received corticosteroids and antibiotics without symptom resolution, and their conditions progressively worsened. After comprehensive clinical evaluation and consideration of patient preference, surgical excision was performed in these cases as well. The volume of excised tissue ranged from 0.75 to 1092.00 cm^3^ (84.75 ± 156.86). Detailed clinical characteristics are summarized in **Table 2**.

**Table 2:** Baseline characteristics of the cases.

Variable	Total (n=84)	Percentage (%)
Obstetric History		
YES	81	96.4
NO	3	3.60
Immunological Diseases		
YES	3	3.60
NO	81	96.40
Use of Psychotropic Medications		
YES	6	7.10
NO	78	92.90
Blunt Trauma to the Breast		
YES	2	2.40
NO	82	97.60

### Microbiological findings

**Figure 1:**
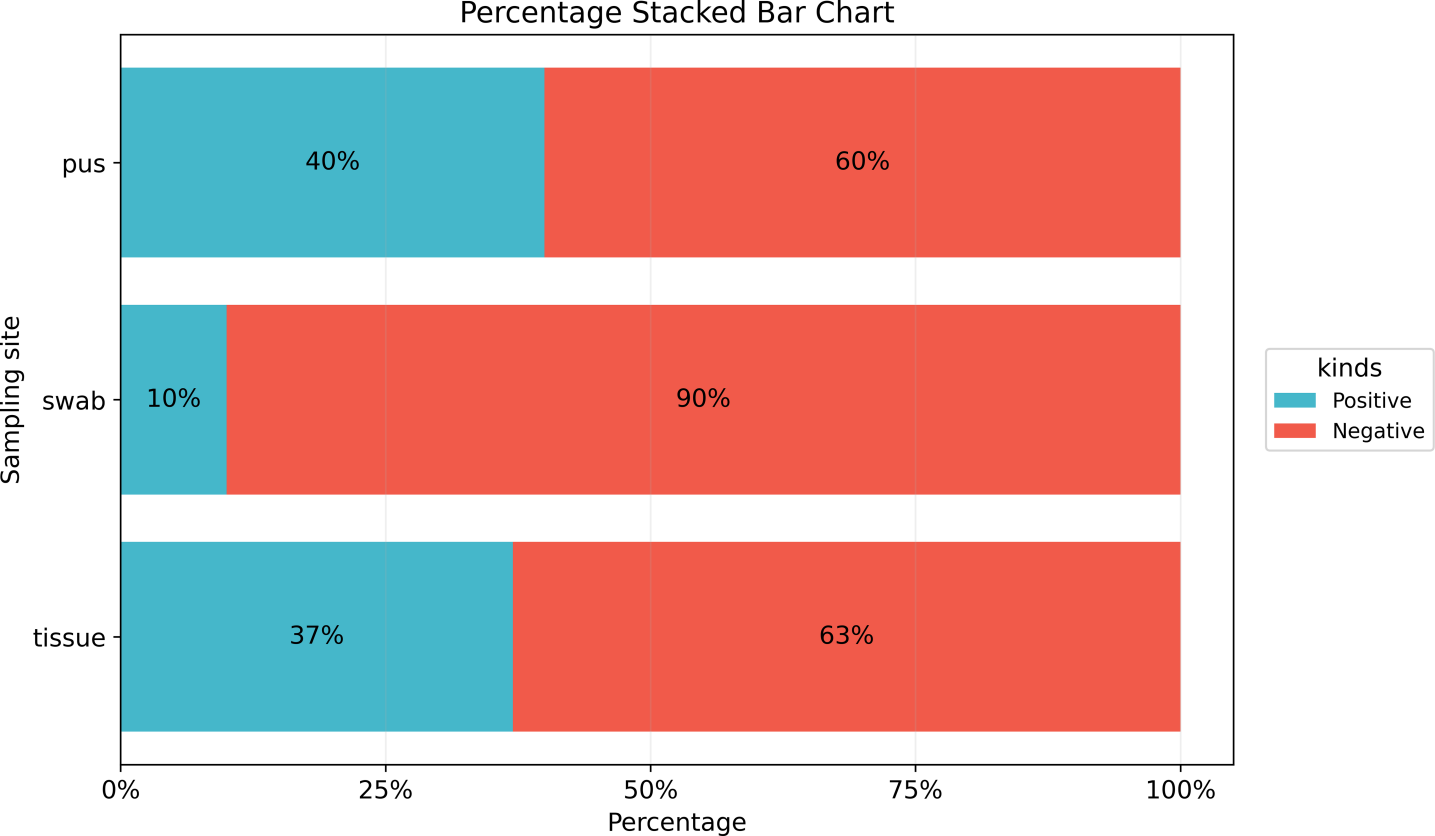
Under different sampling methods for traditional culture, the positive and negative rates vary. The positive rate for skin swab samples is 10% lower, while the positive rates for pus and tissue cultures are similar, at 37% and 40%, respectively.

**Figure 2:**
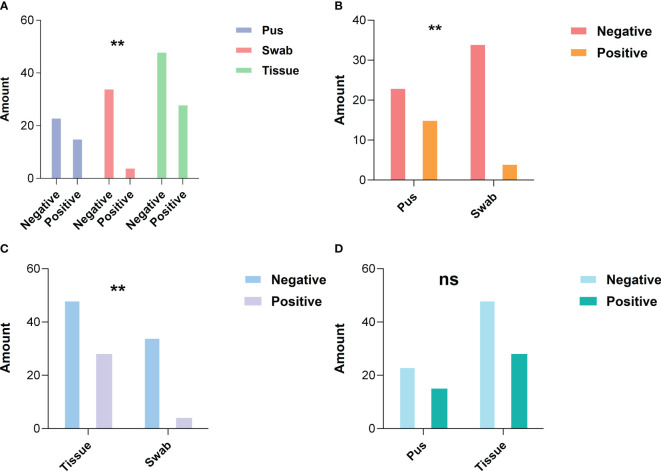
Statistical differences in positive rates between different sampling methods. **(A)** A statistically significant difference is observed in the positive rates among the three sampling methods; **(B)** There is a statistically significant difference in bacterial culture positive rates between pus sampling and skin swab sampling; **(C)** A statistically significant difference in bacterial culture positive rates is found between tissue sampling and skin swab sampling; **(D)** No significant statistical difference is observed in the bacterial culture positive rates between tissue and pus sampling. *P < 0.05, **P < 0.01, ***P < 0.001, ****P < 0.0001 (two-sided Fisher’s exact test).

**Figure 3:**
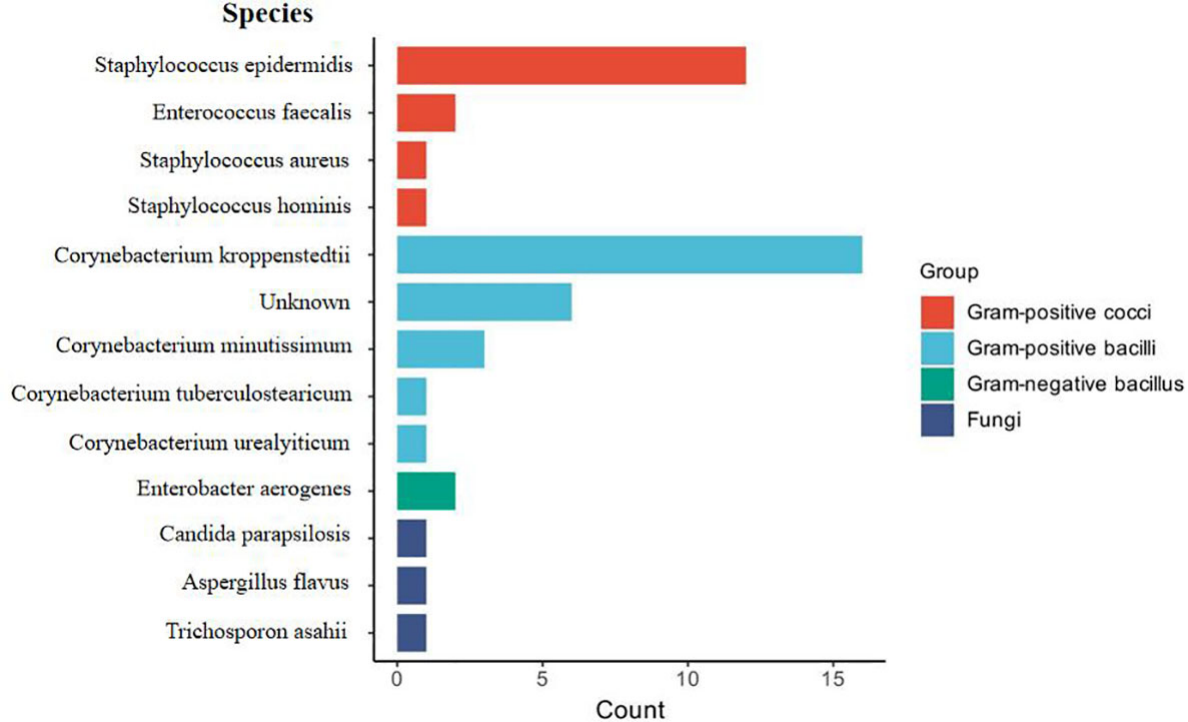
Visual analysis of all bacterial culture results. Statistical analysis of bacterial species identified through traditional culture methods.

All enrolled patients underwent bacterial culture, resulting in the collection of 152 samples. The overall positivity rate was 30.9%, while the negativity rate was 69.1%. The samples comprised 38 purulent fluid specimens (25%), 38 skin swabs (25%), and 76 tissue samples (50%). The incubation period ranged from 1 to 14 days (4.55 ± 1.57 days). Statistical analysis revealed that the positivity rate was highest for purulent fluid (40%), followed by tissue samples (37%), while skin swabs had a significantly lower positivity rate (10%) (**Figure 1**). A significant difference was observed between the positivity rates of skin swabs compared to both purulent fluid and tissue samples (P < 0.05), whereas no significant difference was found between purulent fluid and tissue cultures (P > 0.05) (**Figure 2**). Gram-positive bacteria were the most commonly isolated pathogens, with *C. kroppenstedtii* being the most frequent, followed by *Staphylococcus epidermidis* (**Figure 3**).

### Comparison of mNGS and bacterial culture

Pathogen detection using both mNGS and conventional bacterial culture was performed in a subset of 15 patients. The demographic and clinical characteristics of these patients are summarized in **Table 3**. The patients’ ages ranged from 27 to 59 years (32.67 ± 7.95), with disease durations ranging from 20 to 180 days (85.33 ± 60.58) and hospital stays between 5 and 19 days (12.33 ± 3.79). The excised tissue volumes ranged from 2.00 to 155.25 cm³ (61.05 ± 50.75). Clinically, 10 patients (66.7%) were in the complex refractory phase, while 5 patients (33.3%) were in the abscess formation phase.

**Table 3:** Using mNGS to detect the patient’s characteristics and clinical manifestations.

Variable	Total (n=15)	Percentage (%)	Range	Mean ± SD
Age (year)			27-59	32.67 ± 7.95
Length of Hospital Stay (d)			5-19	12.33 ± 3.79
Duration of Illness (d)			20-180	85.33 ± 60.58
Volume of Tissue Loss (cm³)			2-155.25	61.05 ± 50.75
Obstetric History				
YES	15	100		
NO	0	0		
Immunological Diseases				
YES	0	0		
NO	15	100		
Use of Psychotropic Medications				
YES	1	6.70		
NO	14	93.30		
Blunt Trauma to Breast				
YES	0	0		
NO	15	100		
Nipple Inversion				
YES	1	6.70		
NO	14	93.30		
Erythema Nodosum/Joint Pain				
YES	0	0		
NO	15	100		
Skin Ulcers				
YES	7	46.70		
NO	8	53.30		
Sinus/Fistula				
YES	7	46.70		
NO	8	53.30		
Clinical Staging				
Self-limited	0	0		
Congestive swelling stage	0	0		
Abscess formation stage	5	33.30		
Complex refractory stage	10	66.70		
Position				
Left	8	53.30		
Right	7	46.70		
Bilateral	0	0		
Recurrence				
YES	2	13.30		
NO	13	86.70		

**Figure 4:**
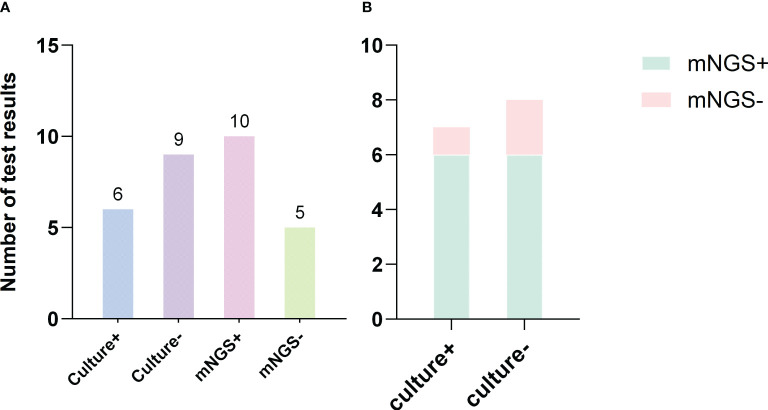
Comparison of mNGS and culture results. **(A)** The number of positive and negative results from bacterial culture and mNGS; **(B)** Comparison of the results between mNGS and bacterial culture.

**Figure 5:**
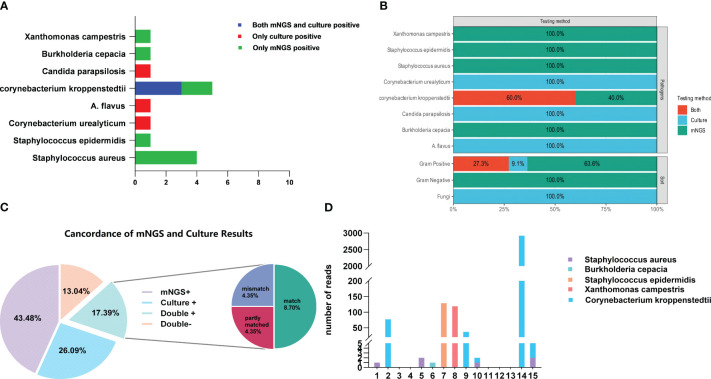
**(A, B)** The types and capabilities of bacterial species detected by bacterial culture and mNGS; **(C)** Comparison of the consistency between bacterial culture and mNGS in detecting bacterial species; **(D)** The number of bacterial sequences detected by mNGS.

The positive detection rate of mNGS (66.7%, 10/15) was higher than that of conventional bacterial culture (40.0%, 6/15). The number of bacterial DNA fragment sequences identified by mNGS is illustrated in **Figure 5D**. Notably, among the 6 patients with negative culture results, mNGS successfully detected pathogens in 4 cases (66.7%). Among the 6 culture-negative patients, 4 (66.7%) had pathogens detected by mNGS. However, the difference in positivity rates between mNGS and culture was not statistically significant (P > 0.05) (**Figure 4**; **Table 4**). *C. kroppenstedtii* was the most frequently detected organism by both methods, followed by Staphylococcus aureus (**Figure 5A**). Notably, mNGS identified a broader spectrum of bacterial species compared to culture, which also detected opportunistic pathogenic fungi (**Figure 5B**). All culture-positive cases involved single-species infections, while mNGS identified two cases of mixed infections.

**Table 4:** Results of multiplicity and sensitivity analyses of six liposomes.

	Result	ID (Infectious Diseases)	NID (Noninfectious disease)		Result	ID (Infectious Diseases)	NID (Noninfectious disease)
mNGS	pos	9	0	Culture	pos	6	0
	neg	3	3		neg	6	3
		Sensitivity	75.00%			Sensitivity	50.00%
		Specificity	100.00%			Specificity	100.00%
		ppv	100.00%			ppv	100.00%
		npv	50.00%			npv	33.33%

Infectious Diseases (ID): GLM diagnosed with the presence of pathogenic microorganisms based on the final clinical diagnosis.

Noninfectious disease (NID):GLM diagnosed with no pathogenic microorganism infection based on the final clinical diagnosis, and attributed to other causes.

Contingency tables formatted in a 2 × 2 manner showing the respective diagnostic performance of mNGS and culture testing for differentiating ID from NID.

Sensitivity was increased by approximately 25% in mNGS compared with culture (75% vs 50%), while specificity remained similar.

Among the 15 patients, 17.39% had pathogens detected by both methods. Complete concordance (same pathogen identified by both methods) was observed in 8.70% of cases, partial concordance (different but overlapping pathogen profiles) in 4.35%, and discordance (mismatched results) in 4.35%. Additionally, 13.04% of patients were negative by both methods (**Figure 5C**).

### Diagnostic performance

**Figure 6:**
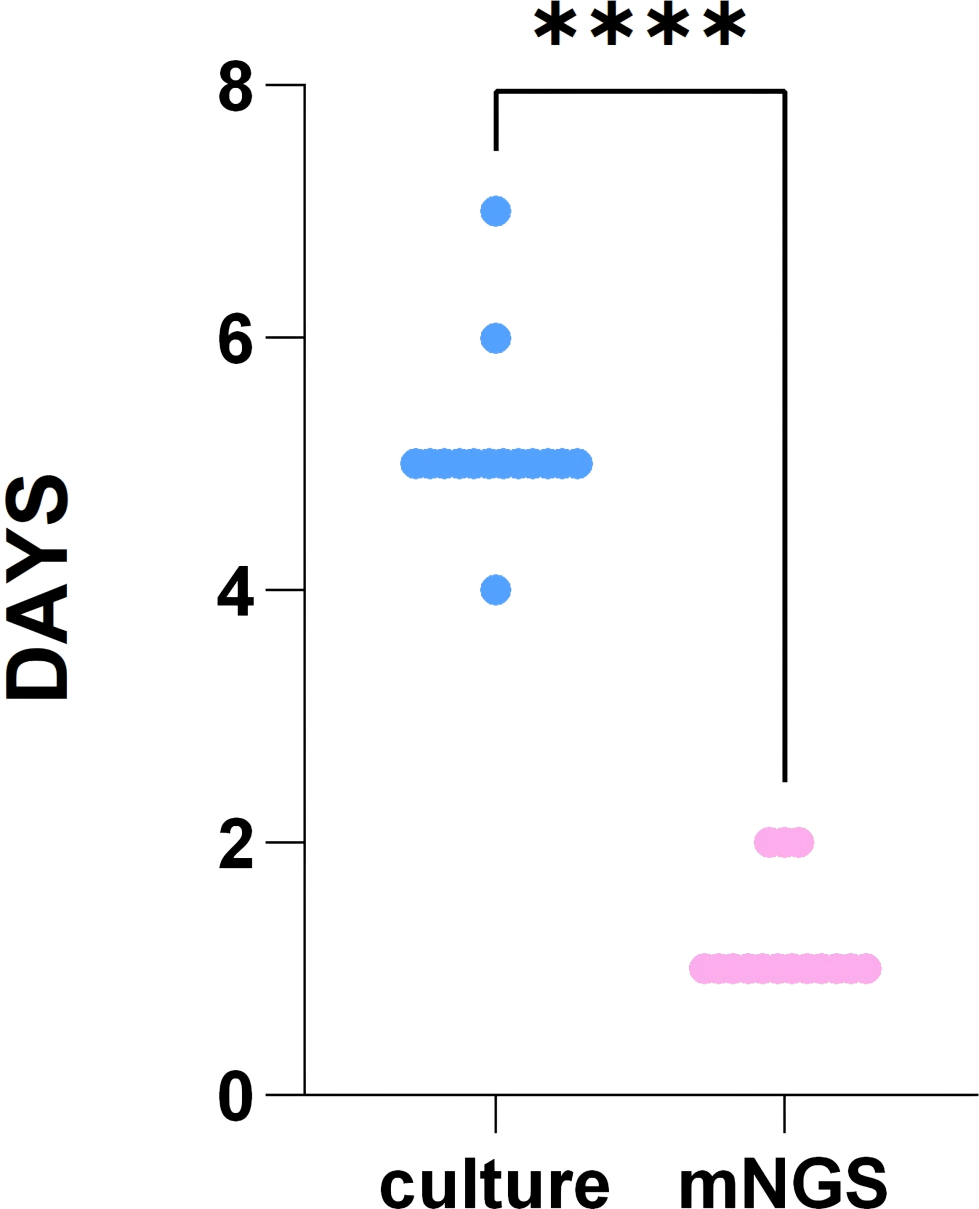
The time cost of the two methods.

The diagnostic performance of mNGS showed a sensitivity of 75.00% (95% CI, 0.47–0.91), specificity of 100.0% (95% CI, 0.44–1.00), PPV of 100.0% (95% CI, 0.70–1.00), and NPV of 50.00% (95% CI, 0.19–0.81). For bacterial culture, the sensitivity was 50.00% (95% CI, 0.25–0.75), specificity was 100.00% (95% CI, 0.44–1.00), PPV was 100.00% (95% CI, 0.61–1.00), and NPV was 33.33% (95% CI, 0.12–0.65) (**Table 4**). The turnaround time for mNGS ranged from 1 to 2 days (1.2 ± 0.41 days), significantly shorter than the bacterial culture time (4 to 7 days, 5.13 ± 0.64 days) (P < 0.05) (**Figure 6**).

### Impact of antibiotic treatment

Among the 15 patients, 12 had received antibiotics within two weeks before sampling(**Supplementary Table S1**). In this subgroup, bacterial culture had a positivity rate of 27.27% (3/12), with *C. kroppenstedtii* not identified in any positive cases. The remaining 9 patients (72.73%) had negative culture results. In contrast, mNGS demonstrated a higher positivity rate of 54.55% (6/12), detecting *C. kroppenstedtii* in 2 cases. Among the 3 patients who did not receive antibiotics, all tested positive for *C. kroppenstedtii* by both culture and mNGS.

## Discussion

### Microbial composition in GLM

This study identified *C. kroppenstedtii* as the predominant pathogen in GLM cases, although infections with other microorganisms were also observed in some patients. In bacterial culture results, *Staphylococcus epidermidis* was identified as the second most common microorganism; however, among patients who underwent both mNGS and culture, *Staphylococcus aureus* was detected as the secondary dominant pathogen. This discrepancy might have resulted from differences in sampling methods, disease stage, or host-related factors. Previous studies have reported that different types of mastitis are associated with distinct pathogens: *S. aureus* is typically linked to acute mastitis, *S. epidermidis* is more common in subacute infections, and *C. kroppenstedtii* is closely associated with GLM (Angelopoulou et al., 2018).

Most of the patients included in our study had prolonged disease duration, recurrent episodes, or complex treatment histories, which may increase the likelihood of mixed infections. Additionally, some patients had received antibiotics before sample collection, either through self-medication or empirical prescriptions, potentially reducing the viable bacterial load. This may lead to false-negative *S. aureus* results in conventional culture. In contrast, mNGS, as a culture-independent technique, detects microbial nucleic acids directly from the sample and therefore retains the ability to identify potential pathogens even when prior antimicrobial exposure has occurred (Fenollar et al., 2006).

*Staphylococcus epidermidis* is a common commensal organism found on human skin and mucosal surfaces, constituting a natural part of the epithelial microbiota (Otto, 2009). Due to its widespread presence, it can easily contaminate clinical specimens during collection or transport. Therefore, its detection in culture requires careful interpretation to determine its clinical relevance (Olearo et al., 2025). In our study, some patients with *S. epidermidis*-positive cultures did present with clinical signs of infection, and therefore, we did not completely consider it as a background contaminant. However, it was not identified as a dominant organism in mNGS results, which may be attributed to its relatively low abundance in tissue samples or the data filtering applied during mNGS analysis, which aims to exclude background flora.

### Sampling strategies in GLM

Accurate identification of pathogenic microorganisms is essential for the effective management of infectious diseases. In clinical practice, the most commonly collected specimens for microbial culture are skin swabs, purulent fluid, and tissue samples. Due to its non-invasive nature and ease of collection, swab sampling is often preferred for initial microbial screening. Some studies have even reported no significant difference in pathogen detection between swab and biopsy samples obtained from the same site (Haalboom et al., 2018; Haalboom et al., 2019). However, our analysis of GLM patients revealed that the positivity rate of skin swabs was significantly lower compared to purulent fluid and tissue samples (10% vs 40% vs 37%, respectively), which showed similar rates, indicating that purulent fluid and tissue samples could be more reliable for pathogen detection in GLM, which also aligns with previous studies indicating that, in soft tissue infections, culturing excised tissue or aspirated purulent material yields higher microbial recovery than swab specimens. The 2020 Delphi consensus also recommends aspirating purulent fluid for microbiological diagnosis (Lipsky et al., 1990; Soriano et al., 2020).

Moreover, studies on GLM have shown that *C. kroppenstedtii* is consistently more abundant in purulent fluid than in tissue, suggesting that this organism may be a key driver of abscess formation (Saraiya and Corpuz, 2019; Chen et al., 2023). Therefore, based on our data and previous research, purulent fluid for pathogen detection is recommended for pathogen detection in GLM, offering both ease of collection and reliable diagnostic value, often comparable to or greater than that of tissue specimens.

### Diagnostic advantages of mNGS

mNGS can detect a wide range of pathogens, with reported sensitivity and specificity varying between 36% and 100%, depending on the disease type and sample characteristics (Miao et al., 2018). The culture of *C. kroppenstedtii*, the main pathogen in GLM, is often challenging due to its stringent growth requirements and slow proliferation rate (Taylor et al., 2003; Bi et al., 2021). Unlike conventional bacterial culture, mNGS directly sequences microbial nucleic acids (DNA and/or RNA) from clinical samples, allowing for more comprehensive detection. In our study, the sensitivity of mNGS for pathogen detection in GLM was 66.7%, higher than the 40% sensitivity observed with bacterial culture, and in patients who received antibiotics before testing, the sensitivity of mNGS (54.55%) remained significantly higher than that of culture (27.27%), supporting the significance of mNGS despite antibiotic exposure, which aligns with findings from other infectious disease studies (Miao et al., 2018). When evaluated against the composite gold standard, the sensitivity of mNGS was 75%, compared to 50% for bacterial culture, confirming the use of mNGS in GLM, especially for identifying mixed infections and optimizing antibiotic therapy. Thus, accurate identification of pathogens in GLM is essential for guiding treatment, and our findings support mNGS as not only having a higher positivity rate but also a broader range of pathogens.

### Antibiotic response of *C. kroppenstedtii* in GLM

Previous studies have shown that C. *kroppenstedtii* is generally susceptible to rifampicin, trimethoprim-sulfamethoxazole, linezolid, and vancomycin, but commonly resistant to β-lactam antibiotics (Dobinson et al., 2015; Zhang et al., 2023), and according to clinical consensus guidelines, prioritizing non-β-lactam antibiotics for antimicrobial therapy in suspected GLM cases is recommended (Yuan et al., 2022). However, in our study, 48 patients (57.14%) received β-lactam antibiotics, resulting in a recurrence rate of 14.58%, while 34 patients treated with non-β-lactam antibiotics had a higher recurrence rate of 20.59%.

Among the 15 patients who underwent mNGS testing, 12 had previously received about a 2-week course of levofloxacin. Despite this treatment, 6 patients still exhibited active infection, including 2 with *C. kroppenstedtii* and the others primarily infected with Gram-positive cocci. This indicates that monotherapy with levofloxacin may not be sufficient for eradicating *C. kroppenstedtii*. Following surgical intervention and postoperative cephalosporin therapy, all patients achieved initial clinical remission, with only 2 recurrences (13.33%) observed during follow-up. Furthermore, the antimicrobial susceptibility of isolated strains should be considered when designing targeted therapy, particularly given the genetic diversity observed among *C. kroppenstedtii* isolates. The use of mNGS for antimicrobial resistance prediction could complement conventional susceptibility testing, facilitating the selection of effective antibiotics, especially in cases where empirical therapy has failed. Thus, integrating pathogen identification with resistance profiling is essential to guide individualized antimicrobial strategies and optimize clinical outcomes.

These findings suggest that cephalosporins may be effective in managing GLM despite previous reports indicating β-lactam resistance in *C. kroppenstedtii*. One possible explanation is that complete surgical excision reduced the bacterial load, enhancing the efficacy of antibiotic treatment. Additionally, recent studies have shown that *C. kroppenstedtii* exhibits considerable genetic diversity, including variations in antimicrobial susceptibility. Some strains are resistant to quinolones, while others remain susceptible to β-lactam antibiotics (Fernández-Natal et al., 2016; Luo et al., 2022; Xiao and Zhao, 2024; Zeng et al., 2024). Moreover, considering that numerous studies have identified *C. kroppenstedtii* as a significant predictor of GLM recurrence, with infected patients exhibiting a 2.16- to 2.64-fold increased risk of relapse (Tariq et al., [[NoYear]]; Tauch et al., 2016; Co et al., 2018; Tan et al., 2019), this highlights the importance of identifying specific strains when selecting antibiotics. Herein, our results indicate that a combination of complete surgical excision and appropriate antibiotic therapy, including cephalosporins, can be effective in managing GLM. Given the variability in resistance patterns among *C. kroppenstedtii* strains, individualized antimicrobial strategies based on accurate pathogen identification are essential to reduce recurrence rates.

### mNGS and AMR prediction

The emergence of antimicrobial resistance (AMR) poses a significant global health challenge, complicating the treatment of infectious diseases and increasing morbidity and mortality (Murray et al., 2022). In this regard, empirical antibiotic use, especially when prolonged or used indiscriminately, can accelerate AMR development. Conventional methods for detecting AMR, such as culture and antibiotic susceptibility testing (AST), are often time-consuming and may fail to identify resistance early. In contrast, mNGS offers rapid and comprehensive detection of resistance genes, making it a promising alternative as it may not only identify pathogens but also predict resistance by analyzing genomic data. Recent studies have demonstrated that AMR profiles predicted by mNGS closely align with conventional AST results. For instance, in pediatric pneumonia cases, mNGS demonstrated greater sensitivity in detecting carbapenem resistance than cephalosporins (Gan et al., 2024). Similarly, machine learning models based on mNGS data accurately predicted resistance in Acinetobacter baumannii (96% accuracy) (Hu et al., 2023) and *Pseudomonas aeruginosa*, achieving high predictive performance (AUC > 0.8) (Cao et al., 2024). These findings highlight the potential of integrating mNGS with computational tools for accurate and early resistance prediction, especially in infections involving multidrug-resistant or fastidious pathogens. Given these advantages, integrating mNGS-derived genomic data with antimicrobial resistance prediction tools, supported by conventional *in vitro* testing, could enhance diagnostic accuracy in GLM and facilitate individualized antimicrobial therapy, thereby optimizing treatment outcomes and minimizing the risk of resistance.

### Non-antibiotic therapies for GLM

Management of GLM remains debated, particularly regarding the roles of steroids, immunosuppressants, and surgery. Current guidelines recommend initiating treatment with local or systemic corticosteroids, reserving surgery for refractory cases. However, at our center, all GLM patients underwent surgical treatment, achieving a remission rate of 82.14% and a relapse rate of 17.86%.

A meta-analysis suggested that combining surgery with steroids results in higher remission (94.5%) and lower relapse (4.0%) compared to surgery alone (90.6% remission, 6.8% relapse). In contrast, using systemic steroids alone was associated with a lower remission rate (71.8%) and a higher relapse rate (20.9%) (Lei et al., 2017). These findings indicate that surgical intervention, whether combined with corticosteroids or not, consistently achieves high remission and low relapse rates. Notably, the difference in relapse between surgery alone and combined treatment is not statistically significant. Although corticosteroids can be effective, their long-term use poses challenges, including prolonged treatment duration and potential hormone-related complications (Lei et al., 2017; Ma et al., 2020; Ong et al., 2024). In comparison, surgery offers a more definitive resolution, especially when antibiotics fail to control the infection. Therefore, surgical management remains a viable primary option for GLM, particularly when corticosteroid therapy is either contraindicated or ineffective.

### Limitations

The study had several limitations that should be acknowledged. First, selection bias might have been present due to its retrospective design, potentially affecting the generalizability of the findings. Second, patients who underwent core needle biopsy or non-surgical treatment were not included, which may limit the representation of the overall pathogen profile of GLM. Third, due to the rarity of GLM and the absence of standardized diagnostic protocols, some patients did not receive systematic microbiological testing at presentation, reducing the representativeness and applicability of the data. Fourth, only 15 patients underwent paired testing with both mNGS and conventional culture, limiting the assessment of diagnostic performance, a limitation acknowledged in the revised manuscript. Lastly, the composite reference standard used was based on clinical diagnosis and culture results, with final microbial interpretation made by infectious disease physicians and microbiologists, which, combined with the limited sample size, may introduce bias in estimating diagnostic accuracy. Future studies could include larger multicenter cohorts to enhance the validity and reliability of diagnostic evaluations.

## Conclusion

In conclusion, surgery plus antibiotic therapy remains essential for managing GLM, especially in cases with abscesses or fistula formation. Since many patients receive empirical antibiotics before diagnosis, identifying pathogens can be challenging. Herein, our study suggests prioritizing pus collection in symptomatic patients, as it provides reliable microbial evidence. Additionally, using mNGS could improve diagnostic efficiency, reduce testing time, and allow for precise antibiotic adjustments. Although the role of antibiotics for *C. kroppenstedtii* remains debatable, our experience indicates that the empirical use of cephalosporins can be effective. All patients in this study underwent surgical intervention for rapid symptomatic relief and obtained promising outcomes. Thus, we consider surgery the most effective option for achieving prompt and complete remission, though further studies are still needed to establish standardized treatment protocols for GLM.

## Data Availability

The clinical data used in this study contain sensitive patient information and cannot be made publicly available due to ethical and legal restrictions. Access to the data can be made available from the corresponding author upon reasonable request and with appropriate institutional approvals.

## Supplementary material

Supplementary Table 1 Antibiotic Use in 15 GLM Patients Undergoing Both mNGS and Culture Testing. Infectious Diseases (ID): GLM diagnosed with the presence of pathogenic microorganisms based on the final clinical diagnosis. Noninfectious disease (NID):GLM diagnosed with no pathogenic microorganism infection based on the final clinical diagnosis, and attributed to other causes. Pre-admission antibiotic use: Based on prior studies and current clinical guidelines, non-β-lactam antibiotics are generally recommended for the treatment of granulomatous lobular mastitis (GLM). In accordance with this, our institution typically administers levofloxacin empirically during outpatient visits prior to hospitalization, as an initial approach to monitor disease progression.
