# Supplementary material for: Pathogen detection and antibiotic use in granulomatous lobular mastitis: a comparison of mNGS and culture
Source: Front Cell Infect Microbiol. 2025 Jun 3;15:1570776. doi: 10.3389/fcimb.2025.1570776 (PMC12188451; doi:10.3389/fcimb.2025.1570776)
Supplement: Supplementary Table 1 — Antibiotic Use in 15 GLM Patients Undergoing Both mNGS and Culture Testing. Infectious Diseases (ID): GLM diagnosed with the presence of pathogenic microorganisms based on the final clinical diagnosis. Noninfectious disease (NID):GLM diagnosed with no pathogenic microorganism infection based on the final clinical diagnosis, and attributed to other causes. Pre-admission antibiotic use: Based on prior studies and current clinical guidelines, non-β-lactam antibiotics are generally recommended for the treatment of granulomatous lobular mastitis (GLM). In accordance with this, our institution typically administers levofloxacin empirically during outpatient visits prior to hospitalization, as an initial approach to monitor disease progression. [file SupplementaryFile1.docx]

Supplementary Table S1. Antibiotic Use in 15 GLM Patients Undergoing Both mNGS and Culture Testing

| Patient ID | Pre-admission Antibiotic | Route | Duration (days) | Pre-admission diagnosis (ID/NID) | Post-admission Antibiotic(s) | Recurrence:Yes/No |
| --- | --- | --- | --- | --- | --- | --- |
| A | Levofloxacin | oral | 10 | ID | Cephalosporins | No |
| B | Levofloxacin | oral | 13 | ID | SMZ-TMP + Linezolid | No |
| C | Levofloxacin | oral | 11 | ID | Cephalosporins | No |
| D | Levofloxacin | oral | 14 | NID | Prulifloxacin | Yes |
| E | Levofloxacin | oral | 10 | ID | Cephalosporins | No |
| F | Levofloxacin | oral | 13 | ID | Cephalosporins | No |
| G | Levofloxacin | oral | 12 | ID | Cephalosporins+Prulifloxacin | No |
| H | Levofloxacin | oral | 9 | ID | Cephalosporins | No |
| I | None | oral | 0 | ID | Cephalosporins | No |
| J | Levofloxacin | oral | 13 | ID | Cephalosporins | No |
| K | Levofloxacin | oral | 11 | NID | Levofloxacin | Yes |
| M | Levofloxacin | oral | 9 | ID | Levofloxacin+Cephalosporins | No |
| N | Levofloxacin | oral | 12 | NID | Levofloxacin | No |
| O | None | oral | 0 | ID | Cephalosporins | No |
| P | None | oral | 0 | ID | Cephalosporins+Levofloxacin | No |

Infectious Diseases (ID): GLM diagnosed with the presence of pathogenic microorganisms based on the final clinical diagnosis.

Noninfectious disease (NID):GLM diagnosed with no pathogenic microorganism infection based on the final clinical diagnosis, and attributed to other causes.

Pre-admission antibiotic use:Based on prior studies and current clinical guidelines, non-β-lactam antibiotics are generally recommended for the treatment of granulomatous lobular mastitis (GLM). In accordance with this, our institution typically administers levofloxacin empirically during outpatient visits prior to hospitalization, as an initial approach to monitor disease progression.
